# Isolation, characterization and liposome-loaded encapsulation of a novel virulent *Salmonella* phage vB-SeS-01

**DOI:** 10.3389/fmicb.2025.1494647

**Published:** 2025-01-24

**Authors:** Yuhang Luo, Jacques Mahillon, Lin Sun, Ziqiong You, Xiaomin Hu

**Affiliations:** ^1^College of Life Sciences, South-Central Minzu University, Wuhan, China; ^2^Laboratory of Food and Environmental Microbiology, Université catholique de Louvain, Louvain-la-Neuve, Belgium

**Keywords:** *Salmonella enterica*, phage, *Guernseyvirinae*, liposome, intracellular antibacterial effect

## Abstract

**Introduction:**

*Salmonella* is a common foodborne pathogenic bacterium, displaying facultative intracellular parasitic behavior, which can help the escape against antibiotics treatment. Bacteriophages have the potential to control both intracellular and facultative intracellular bacteria and can be developed as antibiotic alternatives.

**Methods:**

This study isolated and characterized vB-SeS-01, a novel *Guernseyvirinae* phage preying on *Salmonella enterica*, whose genome is closely related to those of phages SHWT1 and vB-SenS-EnJE1. Furthermore, nine phage-carrying liposome formulations were developed by film hydration method and via liposome extruder.

**Results and Discussion:**

Phage vB-SeS-01 displays strong lysis ability against 9 out of 24 tested *S. enterica* strains (including the pathogenic “Sendai” and “Enteritidis” serovars), high replicability with a burst size of 111 ± 15 PFU/ cell and a titre up to 2.1 × 10^11^ PFU/mL, and broad pH (4.0 ~ 13.0) and temperature (4 ~ 80°C) stabilities. Among the nine vB-SeS-01 liposome-carrying formulations, the one encapsulated with PC:Chol:T80:SA = 9:1:2:0.5 without sonication displayed the optimal features. This formulation carried up to 10^11^ PFU/mL, with an encapsulation rate of 80%, an average size of 172.8 nm, and a polydispersity index (PDI) of 0.087. It remained stable at 4°C and 23°C for at least 21 days and at 37°C for 7 days. Both vB-SeS-01 and vB-SeS-01-loaded liposomes displayed intracellular antimicrobial effects and could reduce the transcription level of some tested intracellular inflammatory factors caused by the infected *S. enterica* sv. Sendai 16,226 and Enteritidis 50041CMCC.

## 1 Introduction

*Salmonella enterica* belong to the *Enterobacteriaceae* family and are among the most common foodborne pathogens. Based on their pathogenicity, *S. enterica* are classified as Typhoidal and Non-typhoidal serovars (Cheng et al., 2019; Johnson et al., 2018). The former consists of the serovars Typhi, Paratyphi A, B, and C, and Sendai, which show high specificity and low transmissibility but cause severe systemic infections such as typhoid and paratyphoid fever in humans (Lamichhane et al., 2024). The latter consists of more than 2,000 serovars, including Enteritidis or Typhimurium, which are usually zoonotic and have a high transmission risk, mostly causing diarrhoea, gastroenteritis, and even septicaemia in some cases (Fierer and Guiney, 2001; Lamichhane et al., 2024).

*Salmonella* are facultative intracellular bacteria (Kehl et al., 2020). They adhere to intestinal epithelial cells via various types of pili adhesins mediated by Type I Secretion System (T1SS) and Type III Secretion System-1 (T3SS-1) (Bao et al., 2020). The effector proteins secreted by Type VI Secretion System (T6SS) and T3SS-1 allow the bacteria to enter the intestinal epithelium via the invaginated plasma membrane of the host cell (Bao et al., 2020; Li et al., 2023). Under the action of T3SS-2, in conjunction with Type IV Secretion System (T4SS) and T6SS, *Salmonella* - containing vacuoles (SCVs) form, in which the bacteria can evade the immune response (Bao et al., 2020; Liss et al., 2017). Some *Salmonella* destroy SCVs and are released into the cytoplasm where they rapidly replicate, lyse the host cell, and reach the extracellular environment (Ménard et al., 2022). The released *Salmonella* are then engulfed by phagocytes and spread throughout the body via the lymphatic and circulatory systems, causing a wide range of clinical symptoms (Li et al., 2023).

Currently, antibiotics are the main treatment agent to pathogenic bacteria. However, most of the antibiotics have problem entering the cells and are often ineffective to intracellular and facultative intracellular bacteria (Coculescu et al., 2014; Ge et al., 2022; Leegaard et al., 1996; Li et al., 2022b; Wu et al., 2015; Zhou et al., 2023). Moreover, the development of bacterial resistance has increasingly become a serious issue globally (Bearson, 2022; Huemer et al., 2020; Sun et al., 2021) and many countries have initiated antibiotic bans in the food and animal husbandry industry (Guidi et al., 2017; Jia et al., 2022; Patel et al., 2020; Tang et al., 2019). Bacteriophages (phages) are viruses infecting bacteria which can be classified as virulent or temperate, associated with lytic and lysogenic cycles, respectively. The former can be developed as antibiotic alternatives due to their target specificity and safety to animals and humans (Abdelsattar et al., 2022; Monteiro et al., 2019; Vikram et al., 2021). Furthermore, phages can enter eukaryotic cells in a variety of ways, such as phagocytosis by macrophages, and endocytosis by epithelial cells (Sweere et al., 2019). In addition, in rare cases, phages may interact with cell surface polysialic acid and internalized via the endolysosomal route without affecting cell viability (Karimi et al., 2016; Lehti et al., 2017; Sapinoro et al., 2008). They have therefore the potential to control both intracellular and facultative intracellular bacteria. New antibacterial agents and technologies developed with single phage and phage cocktail have become a priority in food and animal husbandry industry (Anand et al., 2020; Kaur and Chhibber, 2021; Mijbel Ali et al., 2021). Some successfully implemented and ongoing clinical trials using phage therapy have also received significant attention (Liang et al., 2023; Wang et al., 2024). Yet, some important issues remain to be resolved such as how to prevent the protein shell of phages from being affected by factors such as pH, enzymes (e.g., protease and nuclease) or ions *in vivo*, or how to prevent phages from being inactivated by immune responses (Briot et al., 2022; Jończyk-Matysiak et al., 2019; Jończyk-Matysiak et al., 2017; Minot et al., 2011; Śliwka et al., 2023; Wdowiak et al., 2022).

Liposomes are self-assembly forms of phospholipids that consist of a head with polar phosphate and a hydrophobic lipid tail. When hydrated, the hydrophobic tail turns outward and the head surrounds the water nucleus, forming spherical vesicles made up of phospholipid bilayers, with a range of particle sizes from 30 nm to greater than 10 μm (Akbarzadeh et al., 2013; Almeida et al., 2020). Liposomes have been widely studied as drug delivery agents and protection carriers due to their various advantages such as safety and biocompatibility (Akbarzadeh et al., 2013; Bangham and Horne, 1964). Phages can be hydrated with liposomes, which act as protective vesicles allowing the phages to avoid unfavorable conditions in natural environment and in the living organism as well, promoting stability (Briot et al., 2022; Cinquerrui et al., 2018; Large et al., 2021; Sercombe et al., 2015). Liposomes also mediate the delivery of phage into cells via endocytosis or lipid fusion (Bozzuto and Molinari, 2015; Takikawa et al., 2020).

In this study, a novel virulent *Salmonella* phage was isolated and characterized. It is classified as a member of the *Guernseyvirinae* subfamily, in the *Jerseyvirus* genus. This phage displays a high specificity against some tested pathogenic isolates of Sendai and Enteritidis and has good temperature and pH stabilities. Furthermore, a phage-carrying liposome formulation with optimal potency and encapsulation rate was developed and showed an improved phage stability and persistence compared to the free virions. The phage intracellular antibacterial effect was also improved.

## 2 Materials and methods

### 2.1 Bacterial strains, cell linen and growth conditions

The 37 bacterial strains used in this study are shown in Supplementary Table S1, including *Salmonella* spp. (*n* = 24), *Enterobacter* spp. (*n* = 9) and *Cronobacter* spp. (*n* = 4). All the bacteria were grown in Luria-Bertani (LB) medium at 37°C. The HeLa cell line was provided by the Microorganisms and Viruses Culture Collection Center (MVCCC), Wuhan Institute of Virology, Chinese Academy of Sciences (China). The cells were cultured in a 25 cm^2^ flask containing 5 mL of Dulbecco’s modified Eagle’s media (DMEM, Gbico, USA) supplemented with 10% heat inactivated foetal bovine serum (FBS, Deary tech, China) at 37°C in a 5% CO_2_ incubator.

### 2.2 Isolation and purification of the phage

Three soil samples were collected from a chicken farm in Zhaoyuan City, Shandong Province, China. The crude phage suspensions were obtained by enrichment-culturing with 2 × LB liquid medium, and then precipitated using 10% polyethylene glycol (PEG) 6,000 and centrifuged at 11,000 rpm for 10 min at 4°C. The precipitate was resuspended with SM buffer 50 mM Tris (hydroxymethyl) aminomethane (Tris-Cl, pH 7.5), 100 mM sodium chloride (NaCl), and 10 mM magnesium chloride (MgCl_2_), and the resuspended suspension was mixed with an equal volume of chloroform. The PEG was then removed by centrifugation (3,000 rpm, 15 min). Finally, the concentrated phage suspensions were obtained by filtrated through 0.22 μm filter membranes (Wan et al., 2021).

A double-layer plate method was used to assay the plaque formation, in which the upper layer was melted 0.5% LB soft agar mixed with 100 μL crude phage suspension and 100 μL logarithmic culture of tested bacteria (OD_600_ = 0.9, 10^9^ CFU/mL). A single clear plaque was picked after overnight incubation at 37°C and resuspended into SM buffer. The phage was further purified via multiple repetitions of the double-layer plate culturing and iteratively selection of a single clear plaque. The phage was named as vB-SeS-01.

### 2.3 Host range analysis of the phage

The vB-SeS-01 suspension was serially diluted into 10^8^ to 10^1^ Plaque-Forming Units (PFU/mL). Five μL of each dilution were spotted on the upper layer containing tested host cells in double-layer plates (de Melo et al., 2019). The sensitivities of the hosts were evaluated by the efficiency of plaquing (EOP), which was calculated by the ratio of the average PFU on a tested host to the average PFU on the reference host (*S. enterica* sv. Sendai 16,226).

### 2.4 Characterization of the phage

To determine the phage one-step growth curve, one mL of the mixed suspension containing exponential growing cells of the host bacteria (OD_600_ = 0.9, 10^9^ CFU/mL) and the phage suspension at a multiplicity of infection (MOI) of 0.001 were transferred into 100 mL LB medium and incubated at 37°C, 220 rpm. An equal volume of the bacterial suspension without phage was used as control. Successive samplings were performed at regular intervals (every 10 min) to determine the phage titers and the OD_600_ values of the host bacteria, with or without phage infection.

Furthermore, the physical and chemical properties of the phage were determined by several separate experiments. Firstly, to determine the pH stability, the pH values of the phage suspensions were adjusted by 10-time dilution into the appropriate buffers: 0.1% (w/v) trifluoroacetic acid (pH 2.0), 50 mM disodium hydrogen phosphate-citric acid (pH 3.0), 50 mM sodium acetate (pH 4.0 and 5.0), 50 mM MES (pH 6.0), 50 mM potassium phosphate (pH 7.0), 50 mM Tris-Cl (pH 8.0), 50 mM glycine (pH 9.0) and 50 mM CAPS (pH 10.0), 50 mM sodium carbonate–bicarbonate (pH 11.0), and 50 mM potassium chloride-sodium hydroxide (pH 12.0 and pH 13.0) (Park et al., 2012). Furthermore, to determine the thermal sensitivity, the phage suspensions were incubated at different temperatures (4, 25, 37, 50, 60, 70, 80, and 90°C) for 1 h based on previous study (Huang et al., 2018), then the phage titers were determined after 1 h incubation at 4°C. In addition, to evaluate the effect of chloroform on phage, a final concentration of 5% chloroform was added into 200 μL of viral solution, and an equal amount of SM buffer was added as a negative control, and incubated at 4°C overnight, followed by the determination of the phage titers as previously described (Li et al., 2022a). All experiments were carried out in triplicate.

### 2.5 Genome sequencing and bioinformatics analysis

The phage genome was extracted as previously described (Wan et al., 2024) and sequenced by Shanghai Personal Biotechnology Co., Ltd. (China) using Illumina Hiseq 2,500 sequencer (San Diego, CA, USA) via the second-generation sequencing technology. After sequence quality evaluation, the qualitative data was assembled using SPAdes-3.5.0 (Bankevich et al., 2012). The coding DNA sequences (CDSs) were predicted by RAST (https://rast.nmpdr.org/), PHASTER (https://phaster.ca/) and proksee (https://proksee.ca/). The genes encoding tRNAs were predicted by tRNAScan-SE (http://lowelab.ucsc.edu/tRNAscan-SE/). The putative function of each CDS was manually validated via NCBI BLAST to the databases including Non-Redundant Protein and SwissProt, and the best match was chosen for functional annotation. The potential virulence factor-related gene(s) were validated via the VFDB (http://www.mgc.ac.cn/VFs/), and resistance-related gene(s) were validated using CARD (https://card.mcmaster.ca/). A phylogenetic tree references the International Committee on Taxonomy of Viruses (https://ictv.global/) selected 20 bacteriophages from 5 families based on the terminase large subunit (Supplementary Table S2) was built by MEGA11 using Neighbor-joining method with the Poisson model at a Bootstrap of 1,000 (Tamura et al., 2021; Switt et al., 2015). The phage genome linear comparison between vB-SeS-01, SHWT1 (Accession No. MT740291.1) and vB_SenS_EnJE1 (Accession No. MN336264.1) was performed using Easyfig v2.2.5 (Sullivan et al., 2011).

### 2.6 Preparation of liposome entrapped phage

A thin-film hydration method was used to prepare the liposome as previously described (Colom et al., 2015). A total of 100 mg of L-*α*-Phosphatidylcholine (PC, Sigma-Aldrich, USA), cholesterol (Chol, Sigma-Aldrich, USA), Tween-80 (Biosharp, China), and stearamine (SA, Sigma-Aldrich, USA) were weighed according to the ratios reported (w:w) in Table 1, and dissolved in 10 mL of chloroform and methanol (v:v = 2:1). The suspension was evaporated in a vacuum rotary evaporator (35 rpm, 40°C) until the organic solvents were completely removed and a lipid film formed on the inner wall of the flask. Then 10 mL of phage suspension (1 × 10^11^ PFU/mL) was mixed with the lipid film, and rotary evaporated (35 rpm, 40°C) for 10 min. The resulting milky-white suspensions were put at room temperature overnight. Then each suspension was tripartite divided and ultrasonic treated at 25°C (40 HZ) for 0, 15 and 30 min, using BX5200HP Ultrasonic cleaner (Cimo, China).

**Table 1:** Characteristics of vB-SeS-01-liposomes with different preparations.

Formulation ratio (PC:Chol:T80:SA)	Ultrasonic time (min)	Phage titer (PFU/mL)	Entrapment efficiency (%)	Size (nm)	Poly dispersity index	Zeta potential (mV)
7:3:2:0.5	0	1.69× 10^11^	73.54 ± 3.48	187.20 ± 5.57	0.095	−2.61 ± 0.18
8:2:2:0.5		2.59× 10^11^	71.65 ± 2.15	173.00 ± 3.20	0.114	−0.95 ± 0.15
9:1:2:0.5		2.48× 10^11^	80.11 ± 8.18	172.83 ± 2.78	0.087	−2.41 ± 0.48
7:3:2:0.5	15	2.50× 10^11^	67.70 ± 5.21	203.37 ± 5.85	0.113	−2.12 ± 0.81
8:2:2:0.5		3.69× 10^10^	63.84 ± 3.24	187.67 ± 5.18	0.130	1.16 ± 0.57
9:1:2:0.5		9.06× 10^10^	69.22 ± 6.51	177.03 ± 4.53	0.090	−2.09 ± 0.20
7:3:2:0.5	30	9.09× 10^10^	64.32 ± 13.68	191.43 ± 7.67	0.096	−0.59 ± 0.57
8:2:2:0.5		1.00× 10^11^	69.02 ± 4.60	205.13 ± 5.35	0.160	−0.02 ± 1.11
9:1:2:0.5		1.97× 10^11^	72.38 ± 1.52	203.60 ± 5.60	0.199	−1.10 ± 0.14

The ultrasonicated or non-ultrasonicated suspensions were successively extruded and filtered with GExtruder-10 mL jacketed liposome extruder (Genizer, USA) through 0.4 and 0.2 μm polycarbonate membranes, 8 times each. The obtained vB-SeS-01-liposome complexes were stored at 4°C.

### 2.7 Characterization of the vB-SeS-01-liposome complexes

The encapsulation efficiency of vB-SeS-01-liposome was evaluated as described (Chadha et al., 2017). 10 μL of vB-SeS-01-liposome preparations were mixed with 490 μL of PBS buffer (Biosharp, China), and then 500 μL of 0.02% Triton X-100 (Biofroxx, German) were added and thoroughly mixed at room temperature for 1 h, followed by centrifugation at 12,000 rpm at 4°C for 30 min. The supernatant was filtered and then the phage titer (E_0_) was determined using the double-layer plating method. Furthermore, 1 mL of vB-SeS-01-liposome preparation was centrifuged at 12,000 rpm at 4°C for 30 min, and the free phage titer (E_1_) carried by the supernatant was determined. The phage entrapment efficiency was calculated as follows:


$$\mathrm{Entrapment}\;\mathrm{Efficiency}\left(\%\right)=\frac{\left(E_{0}-E_{1}\right)}{E_{0}}\times 100\%$$

The Dynamic Light Scattering (DLS) analysis was performed as previously described (Cinquerrui et al., 2018). The diluted phage suspension was put into a common cuvette or an electrode cuvette, and the average particle size, polydispersity index (PDI), and zeta-potential of the vB-SeS-01-liposomes were determined using Zetasizer Nano ZSE (Malvern Instruments, UK).

The morphologies of free phage virion and vB-SeS-01-liposome were observed under the transmission electron microscope (TEM) as described (Chadha et al., 2017). 20 μL of phage suspension (10^9^ PFU/ml) and vB-SeS-01-liposome were dropped on a carbon-coated Formvar films and stained with 2% phosphotungstic acid solution, respectively. After air-drying, the phage virions and liposomes were observed using a Talos F200X G2 TEM (ThermoFisher, USA) at an accelerating voltage of 100 kV.

### 2.8 Stability assay of the vB-SeS-01-liposome complexes

The stability analysis of vB-SeS-01-liposome has been performed as previously described (Colom et al., 2015; Singla et al., 2016b). 100 μL of vB-SeS-01-liposome preparations were resuspended using PBS buffer to 1 mL and kept at different temperatures (4, 23 and 37°C) for 0, 7, 14, 21 and 28 days. Similarly, 100 μL of vB-SeS-01-liposome were replenished to 1 mL with intestinal simulating fluid (Perfemiker, China) and kept at 37°C for 30, 60, 90 and 120 min. The equivalent untreated phage suspension was set as control. The particle size was determined using a Zetasizer Nano ZSE (Malvern Instruments, UK), and the phage titer was determined by a double-layer plating method.

### 2.9 Cytotoxicity test

The cytotoxicity assay was performed on HeLa cells using CCK-8 kit (Cell Counting Kit-8, Med Chem Express, USA) following the manufacturer’s instructions 100 μL of HeLa cells resuspended in DMEM (+10% FBS) with a density of 50 cell/μL were put into each well of a 96-well polystyrene plate and incubated in a CO_2_ (5%) incubator at 37°C overnight. The medium was discarded, and then 100 μL of gradient diluted vB-SeS-01-liposomes (10^7^–10^11^ PFU/mL) or free vB-SeS-01 (100–500 μg/mL) by DMEM (+10% FBS) were added into each well and incubated at 37°C for 24, 48, 72 h. The negative controls were the wells without the addition of phage or liposome, and the blank controls were the wells with DMEM only (+10% FBS). After incubation, 10 μL of CCK-8 reagent were added into each well and incubated at 37°C for 1 ~ 2 h. The absorbance at 450 nm was then measured by SpectraMax iD5 Multimode microplate reader (Molecular Devices, USA). The following equation was used to assess the cell survival rate, in which As, Ab, and Ac indicate absorbance of experimental, blank and negative control wells, respectively:


$$\mathrm{Cell}\;\mathrm{Survival}\;\mathrm{Rate}\left(\%\right)=\frac{\left(As-\mathrm{Ab}\right)}{\left(Ac-\mathrm{Ab}\right)}\times 100\%$$

### 2.10 Evaluation of antimicrobial efficacy *in vitro*

The antimicrobial efficacy *in vitro* of the free and liposome-encapsulated vB-SeS-01 was evaluated as described (Singla et al., 2016a). The *Salmonella* bacterial suspensions (16,226 or CMCC50041) were diluted by DMEM (+10% FBS) and added to the cultured HeLa cells at an MOI = 10 in 12-well polystyrene plates (1 × 10^5^ per well) (NEST, China) in a CO_2_ (5%) incubator at 37°C for 3 h, then the medium containing the bacteria was discarded, and the wells were rinsed with PBS several times. The DEME-diluted free vB-SeS-01 (1 × 10^8^, 2.5 × 10^8^, 5 × 10^8^, 1 × 10^9^ PFU/mL) and vB-SeS-01-liposomes (10, 25, 50, 100 μg/mL) were added to the wells and incubated in the CO_2_ (5%) incubator at 37°C for 1 or 3 h. The cells were then incubated with the addition of 100 μg/mL gentamicin for 30 min, which was removed subsequently by washing with PBS, and then 300 μL of 0.5% Triton X-100 were added into the wells, and the plate was placed in the CO_2_ (5%) incubator at 37°C for 8 min. Finally, the cells were washed and resuspended by PBS. The suspension was then diluted and spread on LB plates and cultured at 37°C for CFU counting.

### 2.11 Transcriptional level assay of cytokines

HeLa cells were infected with *Salmonella* 16,226 and CMCC50041 with an MOI = 10, then the former was treated with 1 × 10^9^ PFU/mL of free vB-SeS-01 and 100 μg/mL of vB-SeS-01-liposome complex, respectively, and incubated for 3 h, whereas the latter only for 1 h due to a higher cell apoptosis caused by *Salmonella* CMCC50041 than 16,226. Controls consisted into cell wells with equivalent phages or liposomes without bacteria, and cell wells with neither phage (or liposome) nor bacteria.

Total RNA was extracted by Trizol (Invitrogen, USA) as previously described (Wang et al., 2017), and the cDNA was synthesized using a reverse transcription kit (Takara-Bio, Japan). The transcript levels of IL-6, IL-8, INF-*γ* and TNF-*α* were detected by real-time (RT) fluorescence polymerase chain reaction (PCR) using the dye method by selecting 2× Taq Pro Universal SYBR qPCR Master Mix (Vazyme, China). GAPDH was used as reference gene. The primers used are shown in Supplementary Table S3. Relative quantitative analysis was performed using the 2^-ΔΔCt^ method (Razzuoli et al., 2017).

### 2.12 Statistical analysis

The data are presented as the mea*n* ± standard error of the mean (SEM) of three independent experiments. Statistical analysis was performed using GraphPad Prism software version 9.0.0 (San Diego, California USA) and IBM SPSS (IBM Corp, Armonk, NY, USA). For evaluation of antimicrobial efficacy, two-way ANOVA and unpaired *Student’s t-test* were used, followed by *post hoc* Tukey’s correction. For evaluation the relative expression levels of cytokines in HeLa cells, the 2^-ΔΔCt^ calculation method was used, setting the cells infected or not infected by bacteria as a fixed factor. The data of the cell wells infected with bacteria and treated with free phage, phage-liposome, and DMEM only were subtracted by the data from those of the cell wells not infected with bacteria whereas treated with free phage, phage-liposome, and DMEM only, respectively. Data were analyzed for intergroup differences using one-way ANOVA and unpaired *Student’s t-test*, with post hoc Tukey’s correction. When *p* < 0.05 (*) or *p* < 0.01 (**), the statistical analysis is considered significant.

## 3 Results

### 3.1 Characterization of vB-SeS-01

**Figure 1:**
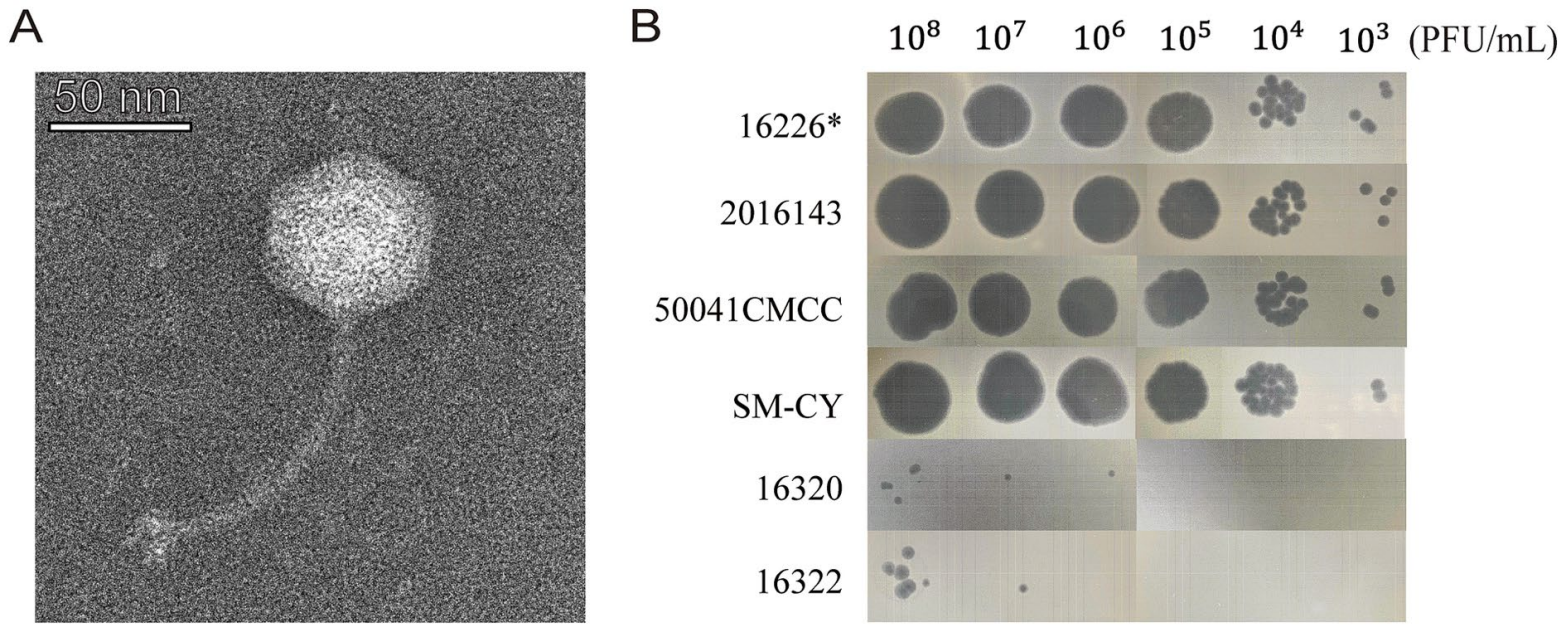
Morphological characteristics of vB-SeS-01. **(A)** Phage morphology under TEM. Scale bar: 50 nm. **(B)** Plaque forming ability. The phage suspensions were serially diluted (10^8^ to 10^3^ PFU/ml) and 10 μL of aliquot were spotted on different strains. *The strain *Salmonella enterica* sv. Sendai 16,226 was used as the propagation host of vB-SeS-01.

Phage vB-SeS-01 has an icosahedral head (57 ± 3 nm) and a long tail (117 ± 13 nm) (Figure 1A), and is active on eight out of the 24 tested *S. enterica,* but not on the nine *E. coli* and four *C. sakazakii* tested. The most sensitive strains were *S. enterica* sv. Enteritidis SM-CY and 50041CMCC, Sendai 16,226 and 2,016,143, on which the phage produces clear plaques and displays the highest plaque formation capacity (Supplementary Table S1; Figure 1B).

**Figure 2:**
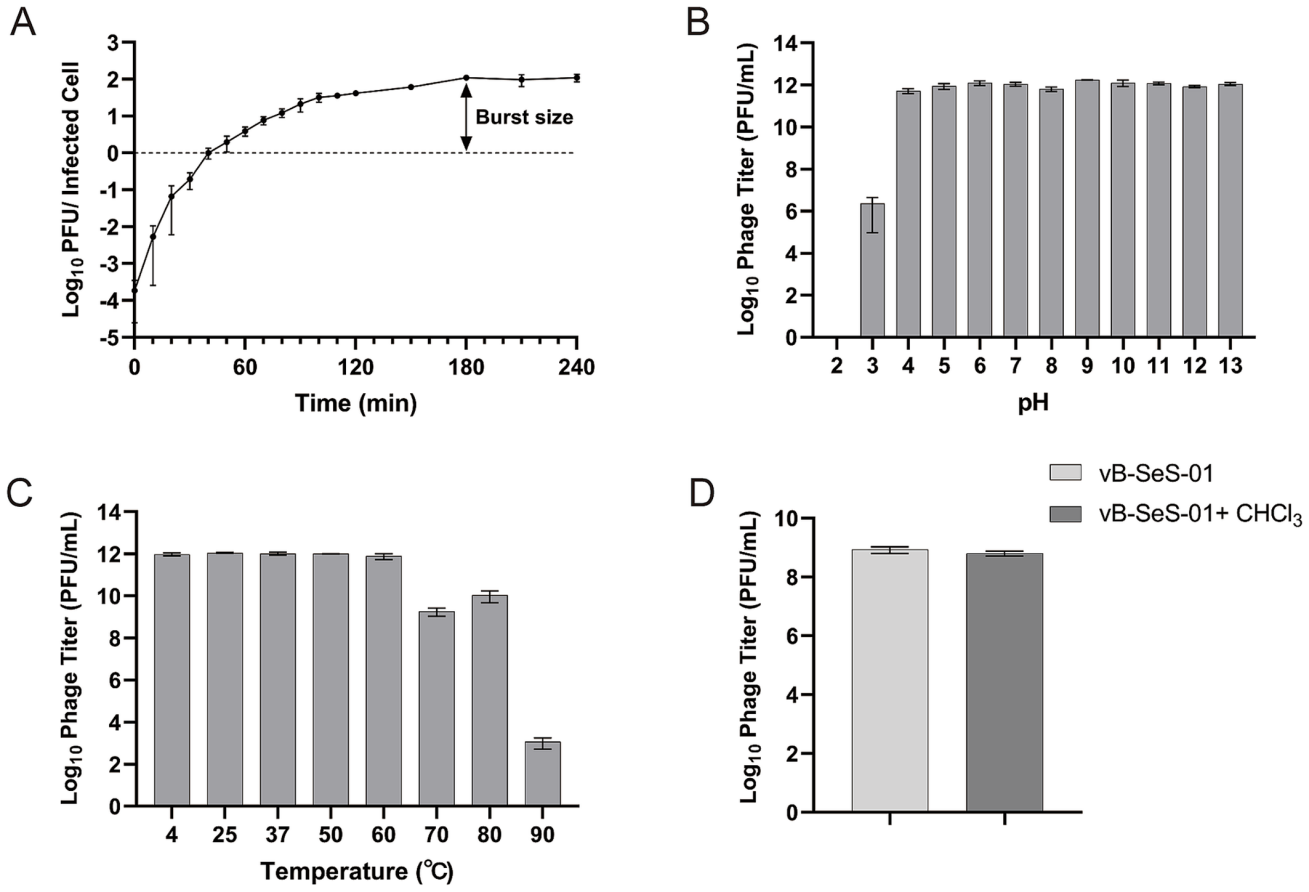
Characterization of vB-SeS-01. **(A)** One-step growth curve. Phage vB-SeS-01 was added to *Salmonella enterica* sv. Sendai 16,226 at a MOI of 0.001. **(B)** pH stability. The phage suspensions were resuspended with reaction buffers at different pH (from 2.0 to 13.0) and kept for 1 h at 4°C. **(C)** Thermal stability. The phage suspensions were pre-treated at different temperatures (4, 20, 30, 37, 50, 60, 70, 80, and 90°C) for 1 h. **(D)** Effect of chloroform on phage. 5% chloroform was added into the phage suspensions. Phage titers were determined after treatment. Data are means ± standard error of means (SEMs) of three parallel samples for each trial.

The one-step growth curve of vB-SeS-01 infecting *S. enterica* sv. Sendai 16,226 was assessed at an MOI of 0.001. It displayed a latent period of 50 min and a burst period of 130 min, with a burst size of 111 ± 15 PFU/cell (Figure 2A) and the titre peaking at 2.1 × 10^11^ PFU/mL at 180 min.

Remarkably, phage vB-SeS-01 showed excellent pH and temperature stability. The titers remained stable from pH 4 to 13 (Figure 2B) and at temperature up to 60°C, still keeping a high titer over 10^9^ PFU/ml at 70 and 80°C (Figure 2C). Also, the phage suspension was not affected by chloroform, indicating the absence of lipid membrane (Figure 2D).

### 3.2 Genetic organization of vB-SeS-01

The genome of vB-SeS-01 is a 43,114 bp circular double-stranded DNA (Accession No. OP494211) with a GC content of 49.7%. A total of 68 CDSs were predicted, categorized into six groups according to their functions (Supplementary Table S4; Supplementary Figure S1), including DNA replication and regulation (*n* = 10), structure (*n* = 18), cell lysis (*n* = 4), transcription (*n* = 1), other functions (*n* = 3), and unknown functions (*n* = 32). No tRNA was predicted and neither integrase, nor virulence factor or drug resistance genes could be predicted.

**Figure 3:**
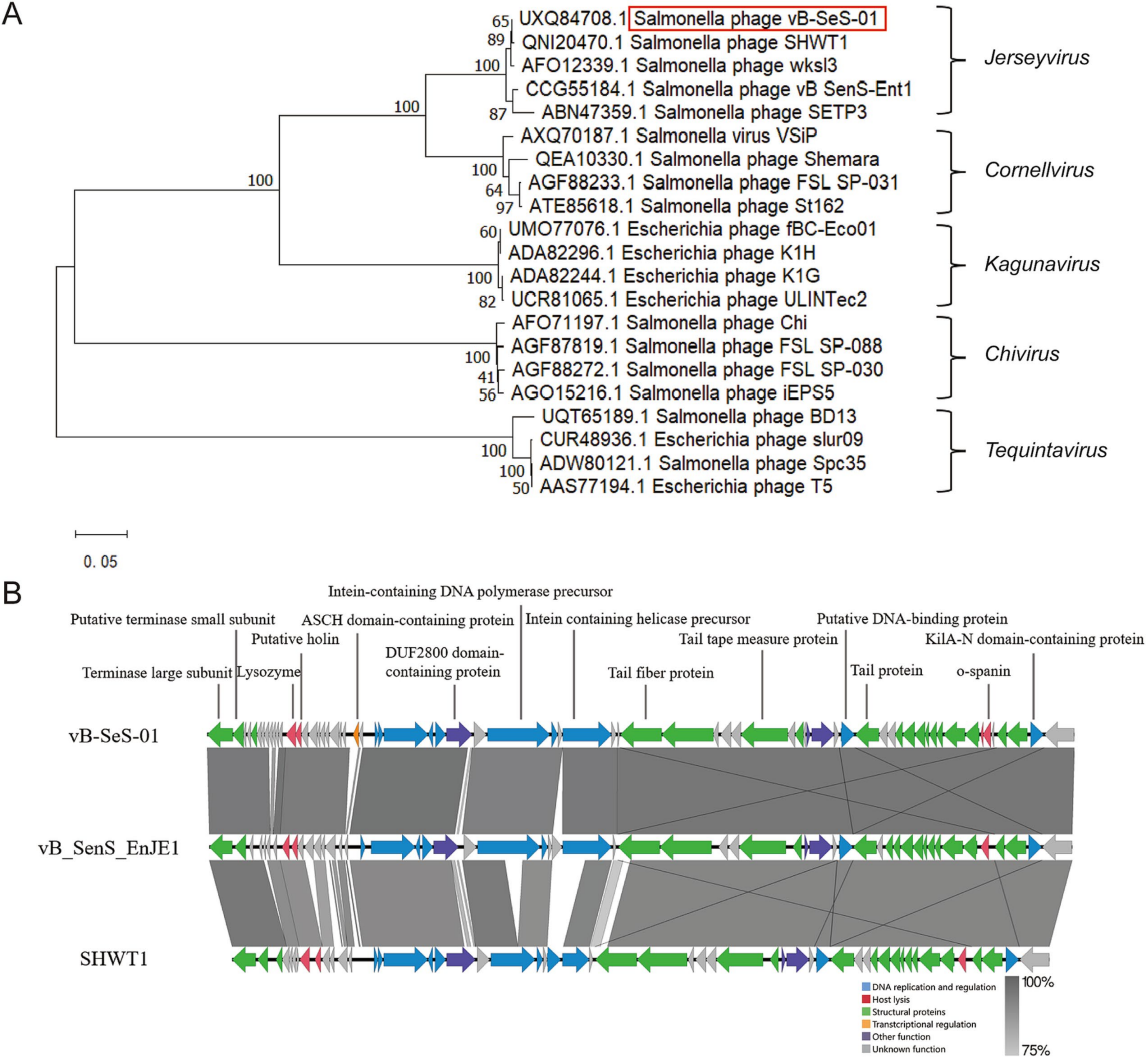
Phylogenetic analysis and genome comparison. **(A)** Phylogenetic tree based on the amino acid sequences of the Terminal Large Subunits (TLS). The dendrogram was constructed using the MEGA X program via the neighbor-joining method, with Poisson model, and tested by a bootstrap of 1,000. **(B)** Genome comparison between vB-SeS-01, vB_SenS_EnJE1 and SHWT1 using Easyfig v2.2.5. Green: structural proteins; red: host lysis; yellow: transcriptional regulation; blue: DNA replication and regulation; purple: other function; grey: unknown function. The grey scale refers to the identities of the amino acid sequences between the compared CDSs.

A dendrogram based on the amino acid sequences of the Terminal Large Subunits (TLS) showed that vB-SeS-01 is a member of the *Guernseyvirinae* subfamily, in the *Jerseyvirus* genus, with phages SHWT1 and vB_SenS_EnJE1 as closest relatives (Figure 3A). The genome of vB-SeS-01 display a coverage of 85 and 96%, and an identity of 93 and 95% to those of SHWT1 (Accession No. MT740291.1) and vB_SenS_EnJE1 (Accession No. MN336264.1), respectively. The main difference among the three phages is that vB-SeS-01 carries four lysis-related genes encoding one endolysin (UXQ84720.1), one holin (UXQ84721.1) and two spanins (UXQ84701.1 and UXQ84702.1) whereas SHWT1 and vB_SenS_EnJE1 carry only three, including one endolysin, one holin and one spanin. In addition, an ASCH structural domain (No. UXQ84730.1) was predicted in phage vB-SeS-01 but absent in SHWT1 and vB_SenS_EnJE1, (Figure 3B) which is thought to potentially function as an RNA-binding domain in connection with co-activation, RNA processing, and prokaryotic translational regulation (Iyer et al., 2006).

### 3.3 Characterization of the vB-SeS-01-liposome complexes

**Figure 4:**
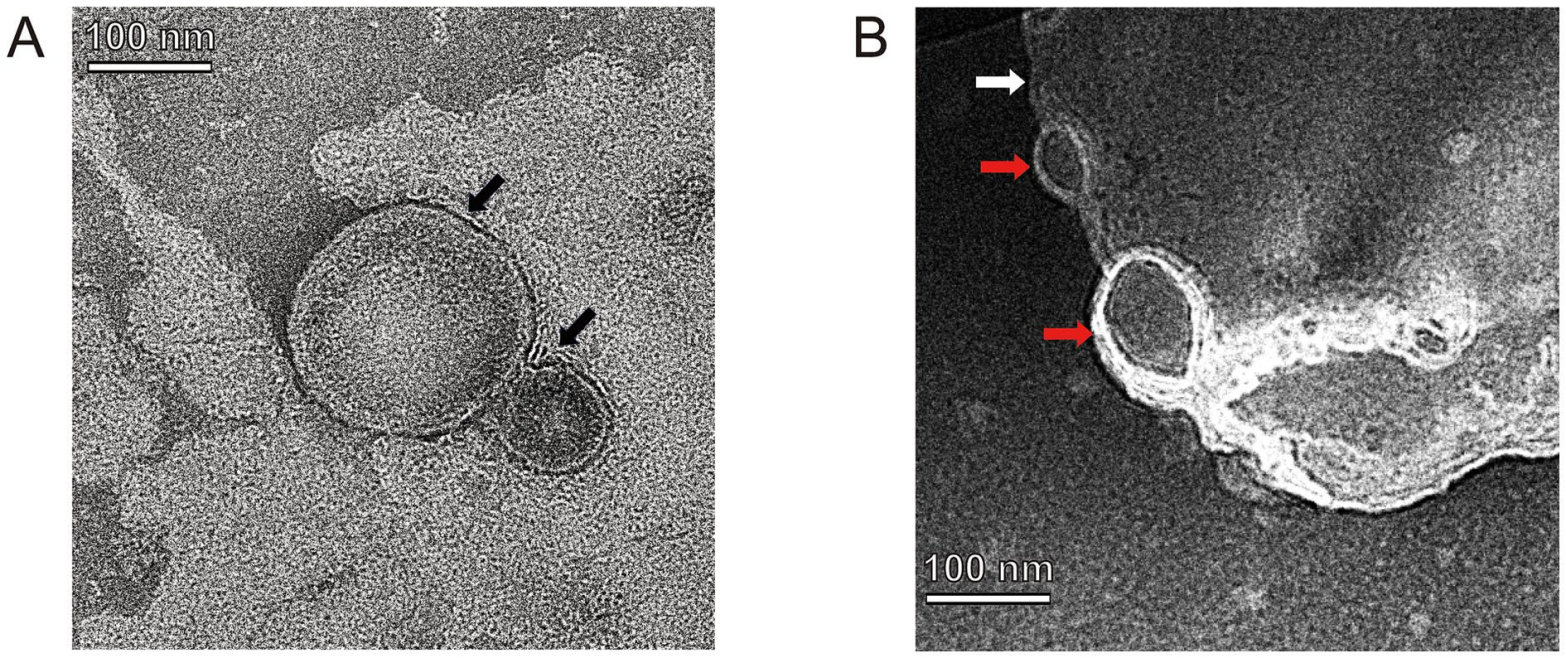
TEM of empty-loaded liposomes **(A)** and phage-loaded liposomes **(B)**. Black arrows indicate empty liposome drops without phage-loaded which displayed as smooth spheres with a diameter of 85.26 and 195.06 nm, respectively. White arrow indicates a phage-loaded liposome which is rough and irregularly, and red arrows indicate phage virions located on its surface.

Nine crude suspensions of vB-SeS-01-liposome complex were produced by adjusting ultrasonic conditions (0, 15 and 30 min) and the ratios of liposomal material (PC: Chol) (Table 1). The three sets without sonication displayed relatively stable titers (about 1.69 ~ 2.59 × 10^11^ PFU/mL), smaller average particle size (172.83 ~ 187.20 nm) and PDI (0.087 ~ 0.114), whereas those sets with sonication displayed varied titers ranging from 3.69 × 10^10^ to 2.50 × 10^11^, larger average particle size (177.03 ~ 205.13 nm) and PDI (0.096 ~ 0.199). Furthermore, the encapsulation efficiency of the sets without sonication was higher than those of the sonicated groups when the same material formulas were used. Phage vB-SeS-01 encapsulated within the PC:Chol:T80:SA = 9:1:2:0.5 liposomes, without sonication displayed the optimal titer (2.48× 10^11^ PFU/mL), particle size (172.83 nm), encapsulation efficiency (80.11%), and PDI (0.087), which are key factors for efficiency, homogeneity and stability. This preparation was therefore retained for the next experiments. Under scanning transmission electron microscopy, the empty liposomes without phage were smooth spheres with average diameters less than 200 nm (Figure 4A), whereas the phage-loaded liposomes were rough and irregularly, with phage virions present on the surface (Figure 4B).

### 3.4 Particle size and stability of the vB-SeS-01-liposome complexes

**Figure 5:**
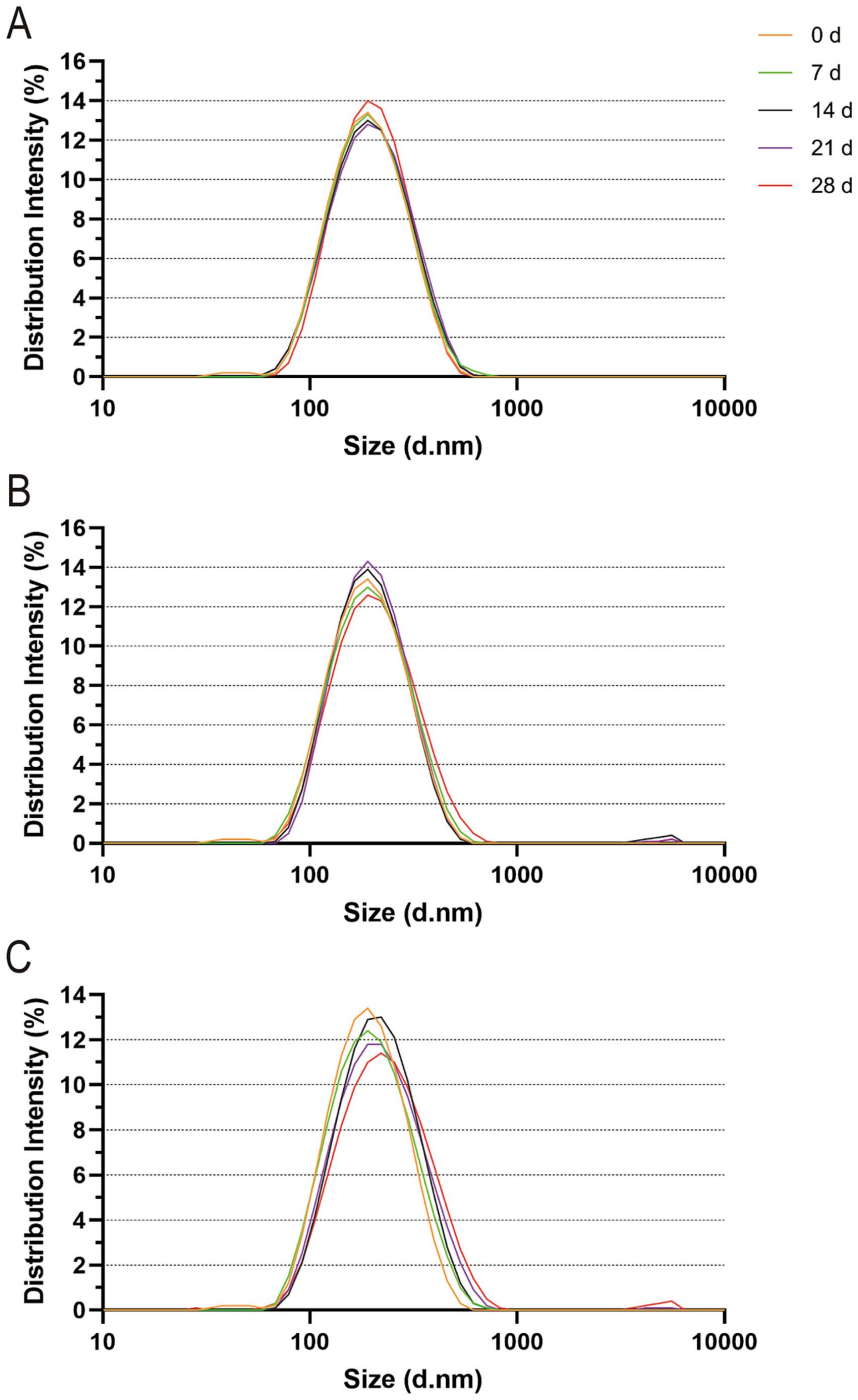
Particle size distribution of vB-SeS-01-liposomes at different temperatures. **(A)** 4°C; **(B)** 23°C; **(C)** 37°C. The different colors refer to the incubation times (in days) as indicated on the top right side. The intensity of the particle size distribution was measured using Zetasizer Nano ZSE (Malvern Instruments, UK), which measure the particle sizes of numerous liposome particles in three sets of 20 cycles and then yield the intensity values based on the distribution (Supplementary Table S5).

The particle size distribution curves of the vB-SeS-01-liposomes were almost identical when kept at 4°C for 0 ~ 21 days (with an average particle size of 177.3 nm), only slightly increased at 28 days (*ca.* 178.2 nm) (Figure 5A; Supplementary Table S5). However, they gradually and slightly increased over time reaching 184.9 nm from 7 ~ 28 days when at 23°C (Figure 5B; Supplementary Table S5). When vB-SeS-01-liposomes were stored at 37°C for 7 days, there was no obvious difference with day 0 (173.9 nm). From day 14 day onwards a decrease in the homogeneity and an increase in the particle size were observed, reaching 187.2, 192.8 and 202.5 nm at 14, 21, and 28 days, respectively (Figure 5C; Supplementary Table S5).

**Figure 6:**
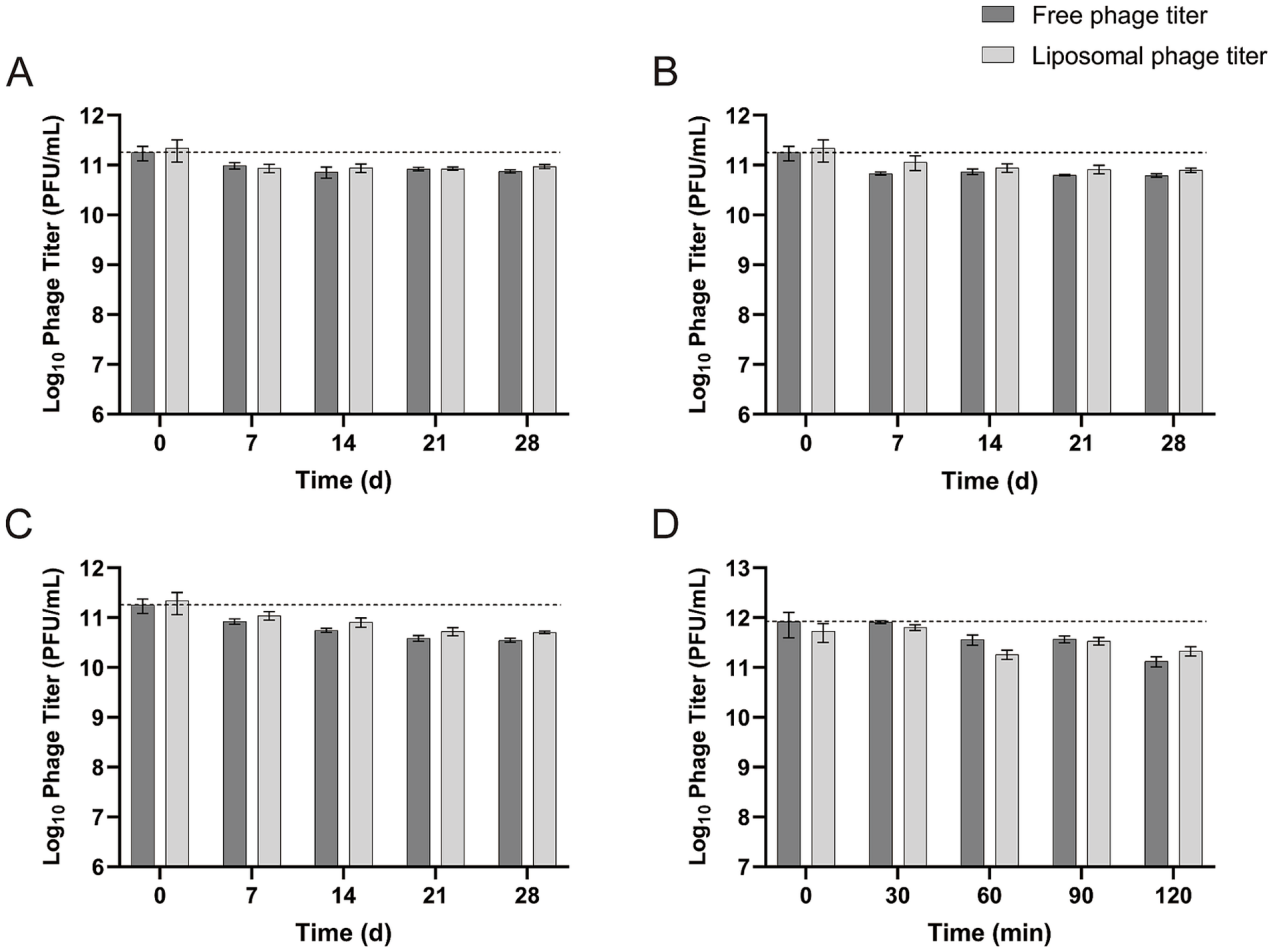
Stability of free vB-SeS-01 and vB-SeS-01-liposomes at different conditions. **(A)** At 4°C; **(B)** 23°C; **(C)** 37°C; **(D)** at simulated intestinal fluid. Data are means ± SEMs of three parallel samples for each trial.

Both free vB-SeS-01 and vB-SeS-01-liposomes displayed a high stability in their titers, decreased by only 0.37 log_10_ at 4°C (Figure 6A), 0.44 log_10_ at 23°C (Figure 6B), and 0.72 log_10_ at 37°C after 28 days of storage (Figure 6C). Of note, the phages carried by liposomes were slightly more stable than the free particles under all temperatures and storage times.

The phage stability of both free and liposome-complexed phages was tested in intestinal simulating fluid up to 120 min. As shown in Figure 6D, the concentration of free vB-SeS-01 and vB-SeS-01-liposomes decreased by 0.35 and 0.20 log_10_, respectively, after being incubated in intestinal simulating fluid for 90 min, and by 0.80 and 0.40 log_10_, respectively, after 120 min of incubation. This indicates a slightly better stability of the vB-SeS-01 phages when complexed with liposomes than as free particles.

### 3.5 Intracellular antibacterial effects of free vB-SeS-01 and vB-SeS-01-liposome complexes

The appropriate dosages of the free vB-SeS-01 and the vB-SeS-01-liposome formulations were determined by verifying the cytotoxic effects on HeLa cell line. Their survival rates were unaffected when treated with up to 10^10^ PFU/mL of free vB-SeS-01 (Supplementary Figure S2A) or 100 μg/mL of the vB-SeS-01-liposomes (carrying 1 × 10^9^ PFU/mL free virions) (Supplementary Figure S2B) during the tested 72 h.

**Figure 7:**
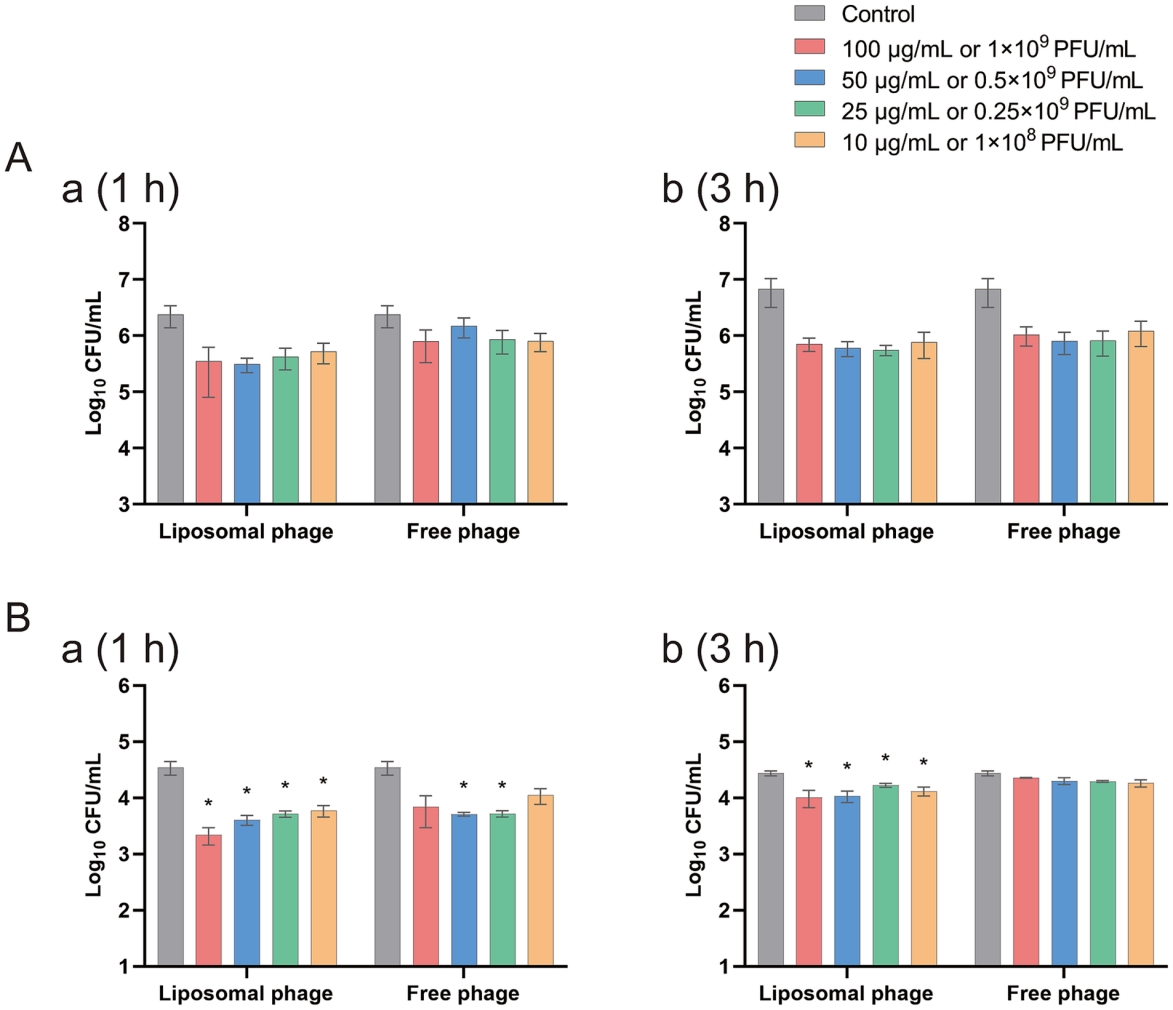
Efficacy of free vB-SeS-01 and vB-SeS-01-liposomes against *Salmonella enterica* sv. Sendai 16,226 **(A)** and Enteritidis CMCC50041 **(B)** in HeLa cells. **(A)** At 1 h. **(B)** At 3 h. Data are means ± SEMs of three parallel samples for each trial (*p < 0.05).

In order to evaluate the antibacterial effect of the free phage virions and the phage-loaded-liposomes on intracellularly colonized *Salmonella*, HeLa cells were pre-infected by *S. enterica* sv. Sendai 16,226 and Enteritidis 50041CMCC at a MOI of 10, and then treated with an equivalent safety dosage of free vB-SeS-01 (10^9^ PFU/mL) *vs.* vB-SeS-01-liposome (100 μg/mL). As reported in Figure 7, in the case of intracellular Sendai 16,226, the reduced number of bacteria by vB-SeS-01-liposome was 0.49 and 0.13 log_10_ more than by free-vB-SeS-01 after 1 and 3 h of sterilization treatment, respectively (Supplementary Figures 7Aa,Ab); whereas for intracellular Enteritis CMCC50041, the reduced number by vB-SeS-01-liposomes was 0.49 and 0.31 log_10_ more than by free-vB-SeS-01 at 1 and 3 h, respectively (Supplementary Figures 7Ba,Bb). The bactericidal effect of vB-SeS-01-liposome on both tested *Salmonella* serovars were better than those of the free phage preparations.

### 3.6 Effect of vB-SeS-01-liposome on cytokine transcription levels

**Figure 8:**
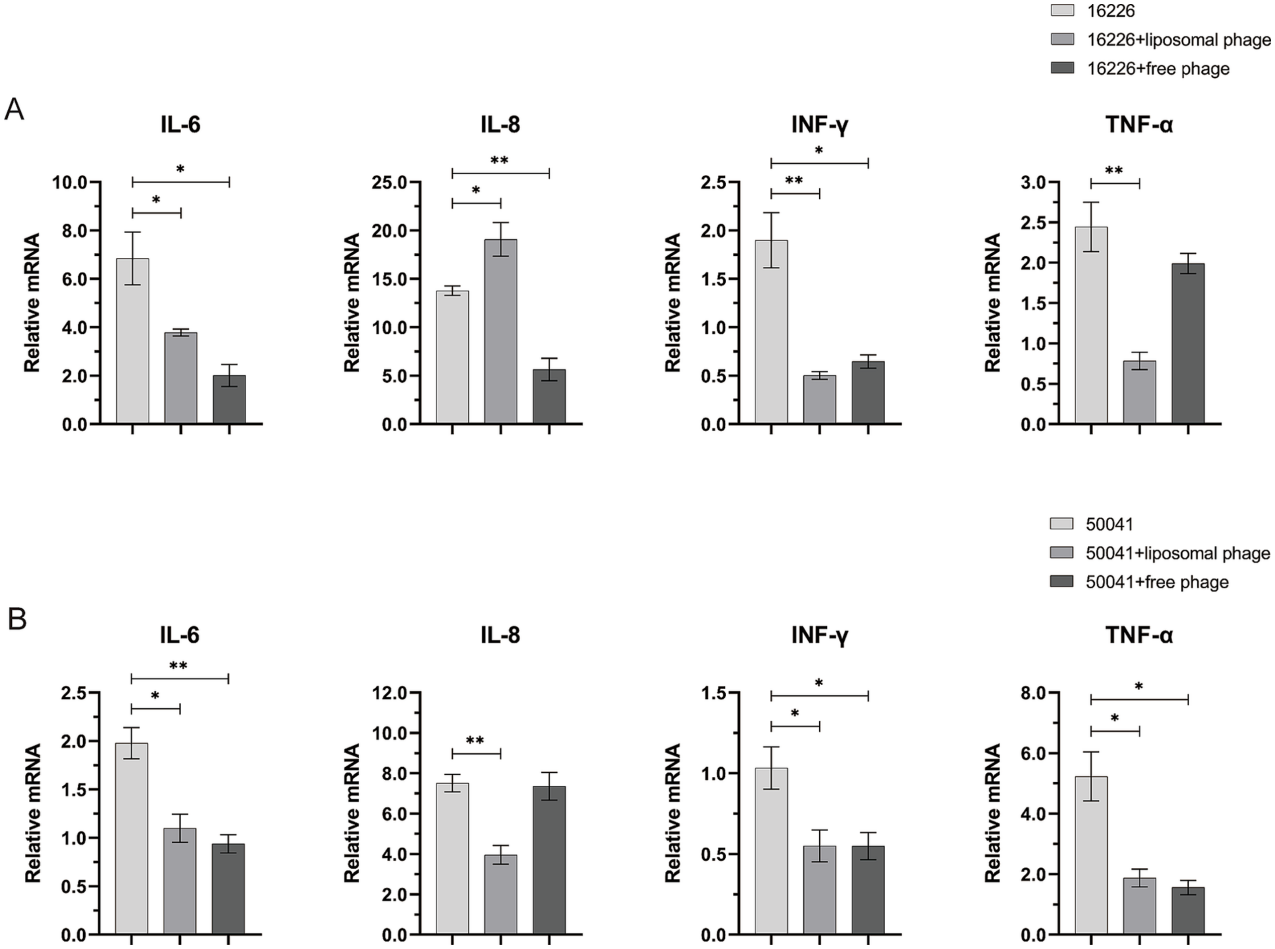
Effects of free vB-SeS-01 and vB-SeS-01-liposomes on relative transcriptional level of cytokine factors infected by *Salmonella enterica* sv. Sendai 16,226 **(A)** and Enteritidis CMCC50041 **(B)**. The free phages were added at a MOI = 10, and the phage-loaded liposomes were added at a concentration of 100 μg/mL with an equal phage titer of 1 × 10^9^ PFU/mL. The treated time was 1 h. Data are means ± SEMs of three parallel samples for each trial (*p < 0.05, **p < 0.01).

The serovar Sendai is human-restricted and elicits enteric fever, whereas serovar Enteritidis has a broad host range (human and animals) and is responsible of gastroenteritis (Feng et al., 2019). As shown in Figure 8A, *S. enterica* sv. Sendai 16,226 infection increased the transcript levels of the intracellular cytokines IL-6 and IL-8 (with a relative mRNA level of 7 and 14 to cells not be infected which were obtained by the 2^-ΔΔCt^ calculation method using GAPDH as an internal reference gene) but not as well on those of INF-*γ* and TNF-*α* (only 1.9 and 2.4 relative to cells not be infected). This is consistent with the clinical observation that the serum levels of INF-γ and TNF-α were relatively low in patients with typhoid fever compared to other salmonellosis symptoms (Gal-Mor et al., 2014). The suppression of their production during the acute phase of typhoid fever has also been reported previously (Butler et al., 1978; Gasem et al., 2003; Girardin et al., 1988; Keuter et al., 1994). The free vB-SeS-01 resulted in a significant reduction of the transcript levels of IL-6 and IL-8, both being reduced to one-fifth of the infection level. Of note, the vB-SeS-01-liposome displayed a relatively lower modulation on IL-6 than the free vB-SeS-01. Moreover, it even resulted an increase of the transcript levels of IL-8 compared to the infection level (Figure 8A).

*Salmonella enterica* sv. Enteritidis CMCC50041 infection increased the transcript levels of the intracellular cytokines IL-8 and TNF-α but not as well on those of IL-6 and INF-γ (Figure 8B). This is corresponding to the result of (Razzuoli et al., 2017), who outlined the different ability of *Salmonella* strains to induce innate immunity and suggested that IL-8 and TNF-α can be used as predictive markers of invasiveness in enterocytes. The vB-SeS-01-liposome and free vB-SeS-01 both displayed similar modulating levels on TNF-α, reducing to one-third of the infection level. However, only vB-SeS-01-liposome complexes decreased the transcript level of IL-8 (Figure 8B).

## 4 Discussion

Reported members of the *Jerseyvirus* genus display a head of 52 ~ 64 nm and a long noncontractile tail of 113 ~ 127 nm (Switt et al., 2015; Tao et al., 2021). The newly isolated *Salmonella* phage vB-SeS-01 is within the range of morphology features of this genus. Furthermore, *Jerseyvirus* phages have a narrow host spectrum infecting specific *S. enterica* serovars (Switt et al., 2015; Tao et al., 2021; Wang et al., 2022). Similarly, vB-SeS-01 displays a narrow host range with strong lysis ability against the tested pathogenic isolates of Sendai and Enteritidis and is highly replicative. Of note, an extra lysis-related gene encoding spanin and an ASCH domain-containing regulation protein that may bind RNA are present in the genome of vB-SeS-01 but absent in its close relatives SHWT1 and vB-SenS-EnJE1. These features may be associated with its specific lytic spectrum. Furthermore, no tRNA-related gene was predicted, indicating that protein synthesis of vB-SeS-01 could be entirely dependent on host tRNAs (Tao et al., 2021). In addition, neither integrase gene nor virulence factor or resistance-related gene presents in the genome of vB-SeS-01, demonstrating the safety of the phage for subsequent applications. Moreover, the phage displays a good pH and temperature stability. The data indicate that vB-SeS-01 has potential for the development of antimicrobial agents against *Salmonella*.

Previous studies suggested that certain fatty acids in lipid-containing phage can inhibit its entry into host bacteria (Muller and Cronan, 1983; Myung and Cronan, 1994; Reinhardt et al., 1978; Sands et al., 1979), which need to be considered when design the formulation of liposome delivery system. Nevertheless, the phage vB-SeS-01 is resistant to chloroform, indicating the absence of a lipid membrane. Phage vB-SeS-01 encapsulated within the PC:Chol:T80:SA = 9:1:2:0.5 liposomes, without sonication displayed the optimal titer. The sonication might result, to some degree, in damaged phage particles (e.g., capsid or tail) and hence reduced phage titer. It might also cause the aggregation or interaction of liposomes, affecting the particle size and PDI. It seemed that a higher ratio of PC: Chol, the better the encapsulation rate on the condition that the phage titres are similar (Table 1). This corresponds to a previous study that suggested that polar head and hydrophobic tail of PC enhance the fluidity of the liposome membrane, making the phage easier to enter the interior of the liposome (Monteiro et al., 2014). However, another study showed that a too high ratio of PC:Chol might lead to a decrease in encapsulation rate (Guimarães et al., 2021). The optimal vB-SeS-01-loaded liposomes carry the virions up to 10^11^ PFU/mL, much more than the T3-loaded liposomes with a maximum titer of 10^9^ PFU/mL (Cinquerrui et al., 2018). No obvious loss of phage titer after encapsulation were observed in our study, a situation similar to the previously reported phage-loaded PEV2 and PEV40 liposomes that only lost 0.66 and 0.40 log_10_, respectively (Leung et al., 2018). The encapsulation rate of vB-SeS-01 reached 80%, close to the best data reported earlier, e.g., 87% of MD-5- and MD-10-loaded, and 92% of KPO1K2-loaded liposomes (Chhibber et al., 2018; Singla et al., 2016b). Furthermore, both the average size of vB-SeS-01-loaded liposomes (172.8 nm) and their PDI (0.087), were smaller than those prepared using microfluidics (average particle size of 207.2 nm and PDI 0.22) (Leung et al., 2018). Interestingly, it has been previously demonstrated that the smaller the PDI, the more uniform the size distribution of each liposome in the formulation. Moreover, it was also shown that the delivery efficiency of the embedded formulations was higher for particle sizes <200 nm (Liu et al., 2021; Sakai-Kato et al., 2019).

The vB-SeS-01-loaded liposomes are stable and remain homogenous at 4°C, 23°C and at 37°C. Only light changes in particle size and virion titer were observed when the samples were stored for 28 d, with an average particle size increased by 5.0–28.6 nm and a titer reduced by 0.37–0.72 log_10_. This is better than some other phage-loaded liposomes previously reported where the average particle size increased by 20 nm at 23°C for 7 days, and 40 and 100 nm at 37°C for 7 and 21 days, respectively (Singla et al., 2016b).

The depletion of free vB-SeS-01 in simulated intestinal fluid can be alleviate when encapsulated. However, both free vB-SeS-01 and vB-SeS-01-loaded liposomes treated with simulated gastric fluid were inactivated in our experiments. This is contrast to the UAB_Phi20-loaded liposomes in simulated gastric fluid that were not completely inactivated but had their titer reduced by 4.2 log_10_ (Colom et al., 2015). The liposome material used in our study are likely susceptible to ions in extremely acidic environments, which might flocculate the liposomes and result in leakage and subsequent inactivation of the encapsulated phage virions. To solve the instability in very acidic environments, surface ion modification might be helpful on vB-SeS-01-loaded liposomes, as previously suggested (Colom et al., 2015).

The vB-SeS-01 phage with a titer of up to 10^10^ PFU/mL had no significant effect on cell activity after 72 h of coexistence with HeLa cells, indicating non-cytotoxic, while the maximum safe dose of the prepared vB-SeS-01-liposome is 100 μg/mL, carrying the vB-SeS-01 virions with a titer of 10^9^ PFU/mL. Different intracellular antimicrobial effects were observed when the vB-SeS-01-liposomes were used against different *salmonella* targets. Generally, the intracellular antimicrobial effects of vB-SeS-01-loaded liposomes were superior to those of free vB-SeS-01 at equivalent titers. This corresponds to a previous study, in which a bactericidal treatment with KPO1K2-loaded liposomes after 3 h brought a reduction of intracellular bacteria by 30.4% vs. the same titer of KPO1K2 by only 4.1% (Singla et al., 2016a). As suggested previously, PC and Chol have good biocompatibility while SA, as a cationic surfactant, can enhance the hydrophilicity of liposomes, helping the delivery of phage into cells through membrane fusion (Guimarães et al., 2021; Németh et al., 2022). Additionally, phages that enter cells via endocytosis can avoid the impact of lysosomes and endosomes (Bozzuto and Molinari, 2015). Thus, the advantage of vB-SeS-01-loaded liposomes over the free phage particles is likely related to their efficient entry into the cells and improved intracellular antimicrobial effect.

INF-*γ* and TNF-*α* are important immune factors, helping the clearance of intracellular *Salmonella* during early invasion (Stoycheva and Murdjeva, 2005). IL-6 is a pro-inflammatory factor, which can mediate the epithelial cell barrier protection displaying anti-inflammatory (Huang, 2021; Shappo et al., 2020) while IL-8 plays a key role in the defence against *Salmonella*, which recruits neutrophils to fight the intruders. However, *Salmonella* can also use this response to affect the gut microbiota and to disrupt the epithelial barrier (Huang, 2012; Razzuoli et al., 2017). The Sendai and Enteritidis serovars exhibit similar O antigens but distinct H antigen formula, activate the inflammasome and the release of cytokines with different levels (Feng et al., 2019). In this work, the infection of *S. enterica* sv. Sendai 16,226 induced higher levels of IL-6 and IL-8 in HeLa cells, while sv. Enteritidis CMCC50041 resulted in higher levels of intracellular IL-8 and TNF-α. The data are in agreement with previous reports that higher levels of TNF-α are often detected in non-typhoidal *Salmonella*-infected patients, and that moderate to high levels of TNF-α, IL-6, and IL-8 are commonly detected in typhoidal and paratyphoidal clinical samples (Gal-Mor et al., 2014). Interestingly, the killing of intracellular *Salmonella* by vB-SeS-01-loaded liposomes and free vB-SeS-01 reduced the transcript levels of most tested intracellular inflammatory factors. Although high levels of IL-8 are induced by both *Salmonella* 16,226 and CMCC50041, however, the unexpected high expression of the IL-8 in the former after treatment by vB-SeS-01-loaded liposomes was observed and remains unclarified. We speculated that (i) a better killing effect of the vB-SeS-01-loaded liposomes than free vB-SeS-01 might result in the releasing of more endotoxins or pathogenic factor(s), which might be particularly inflammatory and lead up to a cytokine storm of certain immune factors as suggested when reaching a particular threshold (Cavaillon, 2018); (ii) the liposome composition might interact with some of the released pathogenic factor(s) of this strain and enhance the production of some cytokines.

The current study has its own limitations toward potential clinical applications. The questions of how to expand the host range of the newly isolated phage vB-SeS-01, how to promote the efficiency of phage/phage-loaded liposome to entry into cells, and how to retain the stability of the vB-SeS-01-loaded liposome both *in vitro* and *in vivo*, remain to be addressed. Furthermore, a key to improving phage therapy will also rely on a better understanding of the differences observed between phage preparations *vs*. phage-loaded liposomes on various cell types.

## Data Availability

The original contributions presented in the study are included in the article/Supplementary material, further inquiries can be directed to the corresponding author.
