# Supplementary material for: Isolation, characterization and liposome-loaded encapsulation of a novel virulent Salmonella phage vB-SeS-01
Source: Front Microbiol. 2025 Jan 24;16:1494647. doi: 10.3389/fmicb.2025.1494647 (PMC11803447; doi:10.3389/fmicb.2025.1494647)
Supplement: Supplementary file 1 [file Data_Sheet_1.DOCX]

Supplementary Materials

# Supplementary Figures and Tables

## Supplementary Figures


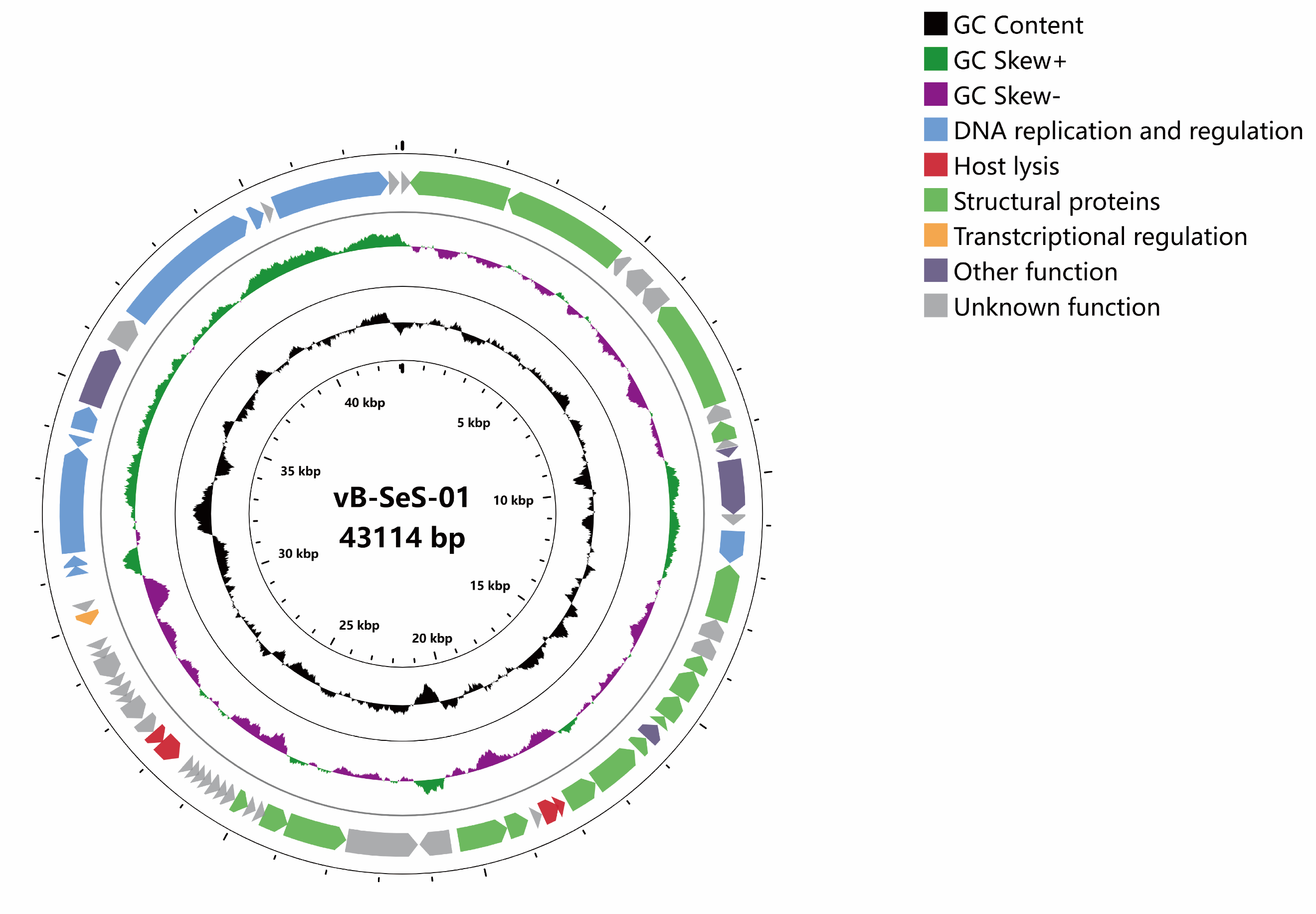


**Supplementary Figure S1.** vB-SeS-01 genome circular map. Green: structural proteins; red: host lysis; yellow: transcriptional regulation; blue: DNA replication and regulation; purple: other function; grey: unknown function.


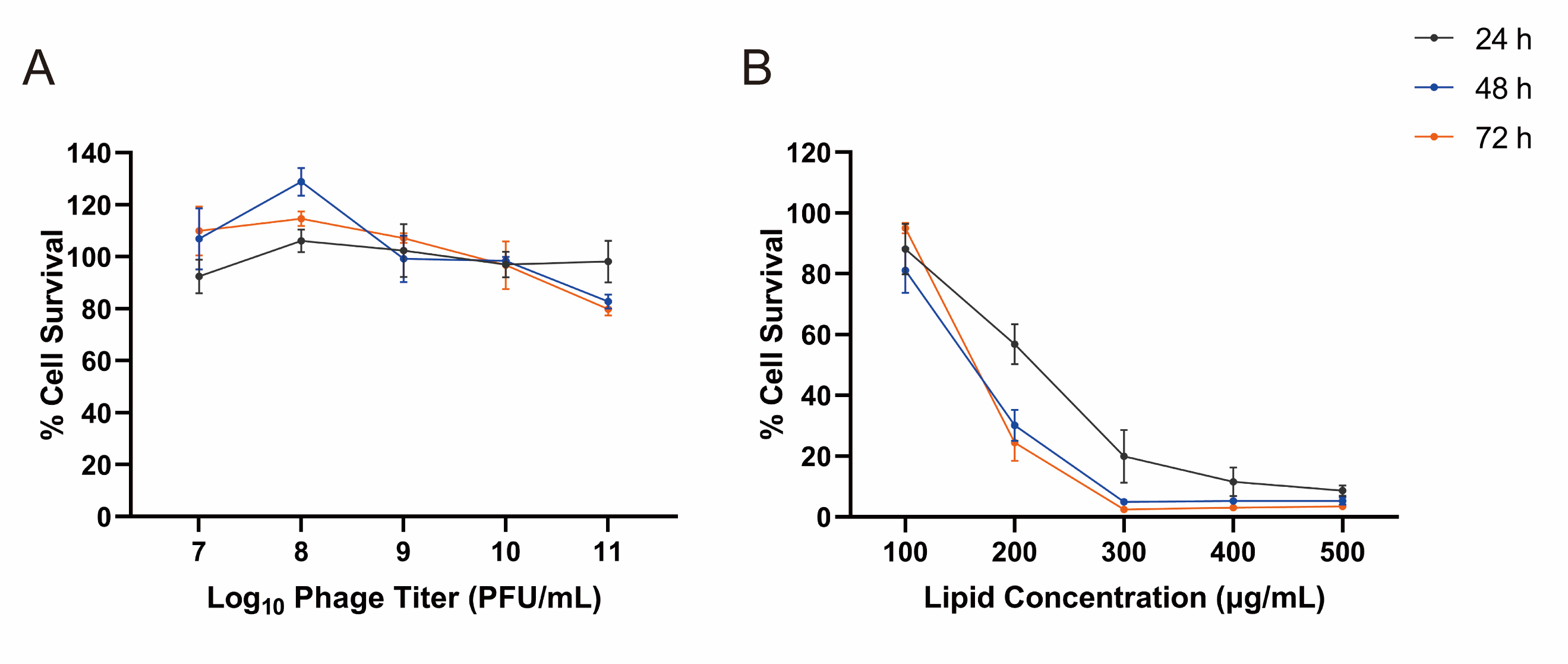


**Supplementary Figure S2.** Effects of free vB-SeS-01 and vB-SeS-01-liposomes on cell viability. (A) Cell viability of Hela cells treated with different titers of vB-SeS-01 for 24, 48 and 72 h; (B) Cell viability of Hela cells treated with vB-SeS-01-liposomes at different lipid concentrations for 24, 48 and 72 h. Data are means ± SEMs of three parallel samples for each trial.

## Supplementary Tables

**Supplementary Table S1.** The host range of phage vB-SeS-01.

| Strains | Characteristics | Origin/Source | Plaque forming ability^a^ |
| --- | --- | --- | --- |
| 16226^b^ | *S. enterica* sv. Sendai | Wuhan Polytechnic University, China | +++ |
| 16320 | *S. enterica* sv. Sendai O9: Ha | Wuhan Polytechnic University, China | ++ |
| 16322 | *S. enterica* sv. Sendai O9: Ha | Wuhan Polytechnic University, China | ++ |
| 2016143 | *S. enterica* sv. Sendai Ⅱ O9: Hg: Ms | Wuhan Polytechnic University, China | +++ |
| 50041CMCC | *S. enterica* sv. Enteritidis | Clinical, China/MVCCC^c^, Wuhan, China | +++ |
| SM-CY | *S. enterica* sv. Enteritidis | Clinical, China/ CDC^d^, Yunnan, China | +++ |
| 16236 | *S. enterica* sv. Sendai | Wuhan Polytechnic University, China | + |
| 16275 | *S. enterica* sv. Sendai O9: Ha | Wuhan Polytechnic University, China | + |
| 16317 | *S. enterica* sv. Sendai O4: Ha | Wuhan Polytechnic University, China | + |
| 16310 | *S. enterica* sv. Equine O4: e,n,x | Wuhan Polytechnic University, China | - |
| 16325 | *S. enterica* sy. Equine O4: e,n,x | Wuhan Polytechnic University, China | - |
| 2016135 | *S. enterica* sv. Equine O4: H-: e,n,x | Wuhan Polytechnic University, China | - |
| 2016139 | *S. enterica* sv. Equine O4: H-: e,n,x | Wuhan Polytechnic University, China | - |
| 16161-1 | *Salmonella,* O7: H7: e,n,x | Wuhan Polytechnic University, China | - |
| 16173-1 | *S. enterica* sv. Equine O4: H-: e,n,x | Wuhan Polytechnic University, China | - |
| 16376 | *S. enterica* sv. Tennessee O7: H829 | Wuhan Polytechnic University, China | - |
| 72-1 | *S. enterica* sv. Paratyphi B O4: H6: 2 | Wuhan Polytechnic University, China | - |
| 74-1 | *S. enterica* sv. Africana O4: H6: e,n,x | Wuhan Polytechnic University, China | - |
| ATCC14028 | *S. enterica* sv. Typhimurium | Clinical, China/ CDC, Yunnan, China | - |
| TA97 | *S. enterica* sv. Typhimurium | CTCC^e^, Wuhan, China | - |
| TA100 | *S. enterica* sv. Typhimurium | CTCC, Wuhan, China | - |
| TA102 | *S. enterica* sv. Typhimurium | CTCC, Wuhan, China | - |
| SM-JXF | *S. enterica* sv. Paratyphi A | Clinical, China/ CDC, Yunnan, China | - |
| SM-YXF | *S. enterica* sv. Paratyphi B | Clinical, China/ CDC, Yunnan, China | - |
| O157 | *E. coli* | Clinical, China/MVCCC, Wuhan, China | - |
| o950 | *E. coli* | South-Central Minzu University, China | - |
| 8099 | *E. coli* | South-Central Minzu University, China | - |
| K12 | *E. coli* | ATCC23716, USA^f^ | - |
| Ssl-1 | *E. coli* | Clinical, China/MVCCC, Henan, China | - |
| Eco2023 | *E. coli* | Clinical, China/MVCCC, Henan, China | - |
| Eco225 | *E. coli* | Clinical, China/MVCCC, Henan, China | - |
| Eco1542 | *E. coli* | Clinical, China/MVCCC, Henan, China | - |
| Ec1616 | *E. coli* | Clinical, China/MVCCC, Henan, China | - |
| ATCC29544T | *C. sakazakii* | ATCC, USA | - |
| CCUG10788 | *C. sakazakii* | CCUG, Sweden^g^ | - |
| CCUG28857 | *C. sakazakii* | CCUG, Sweden | - |
| CCUG28860 | *C. sakazakii* | CCUG, Sweden | - |

^a^ The host range pattern of phage was measured by plaque formation capacity, with ( +++ ) 0.001 < EOP ≤ 1.0, ( ++ ) 0.00001 < EOP ≤ 0.001, ( + ) 0.00001 ≤ EOP, and ( - ) indicating no lysis.

^b^ The strain was used as the propagation host of vB-SeS-01.

^c^ MVCCC, The Microorganisms & Viruses Culture Collection Center, Wuhan Institute of Virology, Chinese Academy of Sciences; ^d^ CDC, Centers for Disease Control and Prevention, China; ^e^ CCTCC, China Center for Type Culture Collection; ^f^ ATCC, American type culture collection; ^g^ CCUG, Culture Collection University of Gothenburg, Sweden.

**Supplementary Table S2.** Information of phages and their Terminal Large Subunits (TLS) used for phylogenetic analysis.

| Family | Phage | TLS (Accession No.) | CDS length (aa) |
| --- | --- | --- | --- |
| *Jerseyvirus* | vB-SeS-01 | UXQ84708.1 | 423 |
|  | SHWT1 | QNI20470.1 | 388 |
|  | vB_SenS-Ent1 | CCG55184.1 | 423 |
|  | SETP3 | ABN47359.1 | 423 |
|  | wksl3 | AFO12339.1 | 423 |
| *Cornellvirus* | FSL SP-031 | AGF88233.1 | 423 |
|  | St162 | ATE85618.1 | 423 |
|  | Shemara | QEA10330.1 | 423 |
|  | VSiP | AXQ70187.1 | 423 |
| *Kagunavirus* | fBC-Eco01 | UMO77076.1 | 416 |
|  | K1G | ADA82244.1 | 416 |
|  | ULINTec2 | UCR81065.1 | 416 |
|  | K1H | ADA82296.1 | 416 |
| *Chivirus* | FSL SP-030 | AGF88272.1 | 691 |
|  | FSL SP-088 | AGF87819.1 | 693 |
|  | Chi | AFO71197.1 | 691 |
|  | iEPS5 | AGO15216.1 | 691 |
| *Tequintavirus* | Spc35 | ADW80121.1 | 438 |
|  | slur09 | CUR48936.1 | 438 |
|  | T5 | AAS77194.1 | 438 |
|  | BD13 | UQT65189.1 | 438 |

**Supplementary Table S3.** Primer sequences for cytokines.

| Primers | Forward primer (5’→3’) | Reverse primer (5’→3’) | reference |
| --- | --- | --- | --- |
| *GAPDH* | GATTTGGTCGTATTGGGCGC | TTCCCGTTCTCAGCCTTGAC | (Wang et al., 2017) |
| *IL-6* | TACCCCCAGGAGAAGATTCC | TTTTCTGCCAGTGCCTCTTT | - |
| *IL-8* | TTTTGCCAAGGAGTGCTAAAGA | AACCCTCTGCACCCAGTTTTC | - |
| *INF-γ* | TGAATGTCCAACGCAAAGCA | CGCTTCCCTGTTTTAGCTGC | (Wang et al., 2017) |
| *TNF-α* | AGCCCATGTTGTAGCAAACC | TGAGGTACAGGCCCTCTGAT | - |

**Supplementary Table S4.** Genome function annotation of vB-SeS-01.

| CDS | Strand | Position | Size (aa)/pI/Mw(D) | Predicted function | Classification |
| --- | --- | --- | --- | --- | --- |
| 1 | + | 31-123 | 30/9.10 / 3069.84 | Hypothetical protein | Unknown function |
| 2 | - | 153-2207 | 684/5.11 / 72855.60 | Tail fiber protein | Structural proteins |
| 3 | - | 2220-4778 | 852/5.27 / 93765.91 | Tail fiber protein | Structural proteins |
| 4 | - | 4769-5032 | 87/5.41 / 9937.18 | Hypothetical protein | Unknown function |
| 5 | - | 5131-5646 | 171/4.46 / 19148.68 | DUF1833 family protein | Unknown function |
| 6 | - | 5643-6143 | 166/5.05 / 18595.90 | Hypothetical protein | Unknown function |
| 7 | - | 6145-8478 | 777/4.72 / 83031.63 | Tail tape measure protein | Structural proteins |
| 8 | - | 8471-8830 | 119/4.69 / 13625.27 | Hypothetical protein | Unknown function |
| 9 | - | 8836-9252 | 138/5.28 / 15877.05 | Tail assembly chaperone | Structural proteins |
| 10 | - | 9242-9364 | 40/9.99 / 4443.15 | Hypothetical protein | Unknown function |
| 11 | + | 9422-9601 | 59/9.70 / 6603.13 | Superinfection immunity protein | Other function |
| 12 | + | 9664-10794 | 376/8.76 / 42485.60 | Oxidoreductase | Other function |
| 13 | + | 10791-11021 | 76/6.05 / 8690.86 | Hypothetical protein | Unknown function |
| 14 | + | 11135-11806 | 223/7.75 / 25752.59 | Putative DNA-binding protein | DNA replication and regulation |
| 15 | - | 11839-13008 | 389/4.63 / 41204.15 | Tail protein | Structural proteins |
| 16 | - | 13008-13427 | 139/4.56 / 15086.11 | Hypothetical protein | Unknown function |
| 17 | - | 13427-13822 | 131/9.69 / 14444.35 | Hypothetical protein | Unknown function |
| 18 | - | 13819-14178 | 119/9.22 / 13128.23 | Tail protein | Structural proteins |
| 19 | - | 14178-14783 | 201/7.99 / 20576.41 | Neck protein | Structural proteins |
| 20 | - | 14786-15295 | 169/4.71 / 17778.89 | Head-tail joining protein | Structural proteins |
| 21 | - | 15299-15487 | 62/4.92 / 7167.96 | Head-tail joining protein | Structural proteins |
| 22 | - | 15524-15874 | 116/4.38 / 12198.54 | Immunoglobulin domain-containing protein | Structural proteins |
| 23 | - | 15886-16173 | 95/9.52 / 9455.75 | Putative head protein | Structural proteins |
| 24 | - | 16234-17283 | 349/4.73 / 37867.41 | Putative coat protein | Structural proteins |
| 25 | - | 17287-17988 | 233/5.78 / 25696.94 | Putative scaffold protein | Structural proteins |
| 26 | - | 18073-18198 | 41/4.51 / 4764.41 | Putative spanin | Host lysis |
| 27 | - | 18182-18568 | 128/9.71 / 14229.00 | O-spanin | Host lysis |
| 28 | - | 18613-18732 | 39/10.91 / 4653.53 | Hypothetical protein | Unknown function |
| 29 | - | 18886-19344 | 152/4.53 / 16419.49 | Neck whiskers protein | Structural proteins |
| 30 | - | 19347-20390 | 347/5.83 / 38722.80 | Head morphogenesis protein | Structural proteins |
| 31 | + | 20556-21206 | 216/5.66 / 24966.42 | KilA-N domain-containing protein | DNA replication and regulation |
| 32 | - | 21237-22715 | 492/4.78 / 54093.48 | 62 kDa structural protein | Unknown function |
| 33 | - | 22728-23999 | 423/5.97 / 47610.59 | Terminase large subunit | Structural proteins |
| 34 | - | 23989-24534 | 181/6.00 / 20523.25 | Putative terminase small subunit | Structural proteins |
| 35 | - | 24524-24655 | 43/4.22 / 4805.36 | Hypothetical protein | Unknown function |
| 36 | - | 24716-24898 | 60/8.19 / 6858.22 | Hypothetical protein | Unknown function |
| 37 | - | 24895-25197 | 100/9.64 / 12181.39 | Putative membrane protein | Structural proteins |
| 38 | - | 25197-25421 | 74/9.34 / 8578.88 | NinH family protein | Unknown function |
| 39 | - | 25418-25567 | 49/7.87 / 5898.93 | Hypothetical protein | Unknown function |
| 40 | - | 25564-25794 | 76/6.26 / 8855.08 | Hypothetical protein | Unknown function |
| 41 | - | 25787-25972 | 61/6.26 / 7316.36 | Hypothetical protein | Unknown function |
| 42 | - | 25969-26124 | 51/9.99 / 5798.64 | DUF2737 family protein | Unknown function |
| 43 | - | 26121-26279 | 52/4.04 / 6060.62 | Hypothetical protein | Unknown function |
| 44 | - | 26276-26461 | 61/7.89 / 6893.97 | Hypothetical protein | Unknown function |
| 45 | - | 26647-27135 | 162/9.64 / 17345.61 | Lysozyme | Host lysis |
| 46 | - | 27113-27403 | 96/9.95 / 10693.90 | Putative holin | Host lysis |
| 47 | - | 27405-27731 | 108/6.54 / 11960.99 | Hypothetical protein | Unknown function |
| 48 | - | 27766-28200 | 144/4.74 / 15586.70 | Hypothetical protein | Unknown function |
| 49 | - | 28206-28337 | 43/10.67 / 5181.12 | Hypothetical protein | Unknown function |
| 50 | - | 28334-28579 | 81/10.69 / 9111.79 | Hypothetical protein | Unknown function |
| 51 | - | 28582-28785 | 67/6.54 / 7406.45 | Hypothetical protein | Unknown function |
| 52 | - | 28782-29279 | 165/4.99 / 18977.17 | Hypothetical protein | Unknown function |
| 53 | - | 29272-29385 | 37/3.58 / 4319.77 | Hypothetical protein | Unknown function |
| 54 | - | 29449-29613 | 54/11.00 / 6262.26 | Hypothetical protein | Unknown function |
| 55 | - | 29983-30258 | 91/8.69 / 11030.69 | ASCH domain-containing protein | Transtcriptional regulation |
| 56 | - | 30274-30477 | 67/9.73 / 7575.77 | Hypothetical protein | Unknown function |
| 57 | + | 31071-31241 | 56/9.38 / 6637.70 | Helix-turn-helix domain-containing protein | DNA replication and regulation |
| 58 | + | 31238-31471 | 77/9.48 / 8617.93 | Putative UvsX-like protein | DNA replication and regulation |
| 59 | + | 31528-33714 | 728/4.96 / 80355.97 | Putative replicative helicase-primase | DNA replication and regulation |
| 60 | - | 33729-33947 | 72/8.80 / 7920.14 | Helix-turn-helix domain-containing protein | DNA replication and regulation |
| 61 | + | 34081-34593 | 170/4.55 / 19245.62 | 13.88 kDa late protein | DNA replication and regulation |
| 62 | + | 34635-35909 | 424/6.35 / 47235.38 | DUF2800 domain-containing protein | Other function |
| 63 | + | 35991-36617 | 208/5.02 / 23471.20 | DUF2815 family protein | Unknown function |
| 64 | + | 36675-39773 | 1032/7.76 / 116146.49 | Intein-containing DNA polymerase precursor | DNA replication and regulation |
| 65 | + | 39860-40147 | 95/9.60 / 10880.58 | Putative restriction endonuclease | DNA replication and regulation |
| 66 | + | 40179-40370 | 63/8.06 / 7084.23 | Hypothetical protein | Unknown function |
| 67 | + | 40408-42837 | 809/8.24 / 90828.03 | Intein containing helicase precursor | DNA replication and regulation |
| 68 | + | 42834-43055 | 73/4.85 / 8722.87 | Hypothetical protein | Unknown function |

**Supplementary Table S5.** Variation of particle size and distribution intensity of vB-SeS-01-liposomes at different temperatures.

*(Provided as an Excel document only)*

# References

Wang, H. W., Zhou, B. H., Cao, J. W., Zhao, J., Zhao, W. P. and Tan, P. P. (2017). Pro-Inflammatory Cytokines Are Involved in Fluoride-Induced Cytotoxic Potential in HeLa Cells. *Biol Trace Elem Res*. 175, 98-102. doi:10.1007/s12011-016-0749-5.
